# 

**Published:** 1897-05

**Authors:** 


### Michigan Dental Association Annual Meeting.

TUESDAY, JUNE 8TH.

2 p. m., Organization; Address of Welcome by Dr. Metcalf, Mayor of Battle Creek, and President's Address with responses.

7:30 p. m., Papers and Discussions.

WEDNESDAY, JUNE 9TH.

9 a. m. The morning will be taken up with Papers and Discussions.

1:30 to 3 p. m., Clinics.

3 p. m., Paper by Dr. J. H. Kellogg, of Battle Creek, on "The Relation of Dental Caries to Indigestion," to be followed by a tour of inspection of the city and a hygienic dinner at the Sanitarium.

8 p. m., Business Meeting. Election of officers.

THURSDAY, JUNE 10TH.

9 a. m., Papers and Discussions. 2 p. m., Clinics. To close with a banquet in the evening.

---

THE *Therapeutic Gazette* says that the oil of cinnamon, 1 to 500, is an anæsthetic when directly applied to mucous membranes.

### Illinois State Dental Society.

The thirty-third annual meeting of the Illinois State Dental Society will be held at Peoria, May 11th to 14th, inclusive, 1897. An excellent program is in the course of preparation. Members are urgently requested to be present. The profession, generally, is cordially invited. A reduced rate of one fare and a third has been granted from all points within the state.

Louis Ottofy, Secretary,
Masonic Temple, Chicago.

### Dental Societies—Directory.

The Dental Register began the publication of the list of the Dental Societies in the United States a number of years ago. This work was commenced at the suggestion and even the urgent solicitation of a number of the leading societies because of the convenience of having such a list for ready reference. This list embraced the name of the society, the time and place of its meetings and the names of its president and secretary, with the address of each. It was promised by many societies that through their secretaries this list should be kept accurate and complete.

For several years this was very well done. Bye and bye, however, this was neglected and inaccuracies in more or less of the particulars was the result. The editor has occasionally made corrections himself, but a great many inaccuracies occured, for which the societies must be held responsible. By a recent effort these statistics have been almost wholly corrected and brought up to date and it is to be hoped from this time on that the officers of the various societies will be sufficiently interested in this matter to send a statement of any changes in their societies to the editor of the REGISTER so soon as they may occur, so that in the futuer this list may be referred to with assurance of its accuracy. A copy of this number of the REGISTER will be sent to the secretary of every Dental Society in this country. If any society neglects or refuses to give this data in the future, it will be dropped from this roll.

### Programme of the Dental and Oral Section of the American Medical Association.

1. Chairman's Address, by Dr. R. R. Andrews.

2. "Resection and Reproduction of the Maxilla," by Dr. G. Lenox Curtis.

3. "Sterilized Roots of Beast's Teeth as Supports for Porcelain Crowns," by Dr. W. E. Walker.

4. "Tumors of the Maxilla," by Dr. W. Knight.

5. "The Influence of Diseased Teeth upon the Adjacent Structures," by Dr. J. Taft.

6. "Etiology and Treatment of Inflammation of the Anterior Pillars of the Fauces," by Dr. Geo. T. Carpenter.

7. "Pathologic Conditions of the Throat and Contiguous Structures during Early Childhood, Prime Factors in the Causation of Irregularities of the Maxilla and Teeth," by Dr. Wm. J. Mills.

8. "Dental Faculties in Medical Schools," by Dr. Richard Grady.

9. "The Need of Dental Instruction in Medical Schools," by Dr. Edward Bramgan.

10. "A Series of Clinical Cases," by Dr. Vida A. Latham.

11. Subject not yet announced, by Dr. G. V. I. Brown.

12. Subject not yet announced, by Dr. Geo. T. Eames.

13. "Cataphoresis (*i. e.*,) The Use of Electricity alone for Obtunding Sensitive Dentine," by Dr. W. A. G. Bonwill.

14. "Maternity," by Dr. A. C. McCurdy.

15. "Pyorrhoea Alveolaris in Mercurial and Lead Poisoning and Scurvy—Paper No. 4," by Dr. E. S. Talbot.

### CONTENTS.

COMMUNICATIONS.

### THE DENTAL REGISTER,

A MONTHLY MAGAZINE DEVOTED TO THE INTERESTS OF THE

**DENTAL PROFESSION.**

*Terms Reduced to* $2.00 *Per Year. Single Copies,* 25 *Cents.*

**EDITED BY PROF. J. TAFT, D.D.S.**

—AND—

**Published by SAM'L A. CROCKER & CO., Ohio Dental and Surgical Depot.**

The volume commences in January and closes in December.

The REGISTER being issued monthly is always fresh, therefore Indispensable to the practitioner who desires to keep posted up with the new inventions and improvements which are daily developed. Neither expense nor effort will be spared to give value and variety to its contents. Articles will be illustrated with well executed engravings whenever they will add to the interest or assist to explanation.

Its extensive circulation and frequency of issue make it one of the BEST DENTAL ADVERTISING MEDIUMS in the United States.

All subscriptions must begin with January or July.

Local or special notices, five lines or less, will be inserted for $3 each insertion; over five lines, 50 cts. per line.

All advertising bills payable in advance, or at furthest, after the issue of the first number containing the advertisement. All transient advertisements to be paid for in advance.

☞CONTRIBUTIONS SOLICITED.

Exclusively Editorial Communications should be addressed to the Editor.

The latest number of the REGISTER may be ordered with or without other goods [illegible] any time, and all letters should be addre[illegible]

